# POU domain motif3 (Pdm3) induces *wingless* (*wg*) transcription and is essential for development of larval neuromuscular junctions in *Drosophila*

**DOI:** 10.1038/s41598-020-57425-9

**Published:** 2020-01-16

**Authors:** Yeon Kim, Kyung-Ok Cho

**Affiliations:** Department of Biological Sciences, Korea Advanced Institute of Science and Technology, 291 Daehak-ro, Yuseong-gu, Daejeon Korea

**Keywords:** Morphogen signalling, Developmental neurogenesis

## Abstract

Wnt is a conserved family of secreted proteins that play diverse roles in tissue growth and differentiation. Identification of transcription factors that regulate *wnt* expression is pivotal for understanding tissue-specific signaling pathways regulated by Wnt. We identified *pdm3*^*m7*^, a new allele of the *pdm3* gene encoding a POU family transcription factor, in a lethality-based genetic screen for modifiers of Wingless (Wg) signaling in *Drosophila*. Interestingly, *pdm3*^*m7*^ larvae showed slow locomotion, implying neuromuscular defects. Analysis of larval neuromuscular junctions (NMJs) revealed decreased bouton number with enlarged bouton in *pdm3* mutants. *pdm3* NMJs also had fewer branches at axon terminals than wild-type NMJs. Consistent with *pdm3*^*m7*^ being a candidate *wg* modifier, NMJ phenotypes in *pdm3* mutants were similar to those of *wg* mutants, implying a functional link between these two genes. Indeed, lethality caused by Pdm3 overexpression in motor neurons was completely rescued by knockdown of *wg*, indicating that Pdm3 acts upstream to Wg. Furthermore, transient expression of Pdm3 induced ectopic expression of wg-LacZ reporter and Wg effector proteins in wing discs. We propose that Pdm3 expressed in presynaptic NMJ neurons regulates *wg* transcription for growth and development of both presynaptic neurons and postsynaptic muscles.

## Introduction

Transcription factors play essential roles by inducing genes during the formation of body plans, organ development, tissue specificity, and generation of diverse cell types. Numerous transcription factors are grouped based on similarity in their sequences and domain structures. Pituitary-specific positive transcription factor 1, Octamer transcription factor-1, Uncoordinated-86 domain (POU) transcription factors^1^ belong to a subfamily of homeodomain transcription factors, and are highly conserved in all metazoans^2–5^. POU domain consists of two DNA binding domains, POU homeodomain and POU specific domain, and these two domains are linked by a flexible linker^5,6^. Based on sequence homology of the POU domain and the linker, POU proteins are grouped into six classes^2,4,5^. POU proteins are often expressed in spatiotemporally restricted patterns during development, implying that they may be specialized for differentiation of specific cells or tissues by activating required signal transduction pathways^5^.

The class VI *Drosophila* POU domain motif 3 (Pdm3) protein is reported to function in olfactory receptor neurons (ORNs) by regulating olfactory receptor gene expression and axon targeting, and in ring (R) neurons by regulating the development of ellipsoid body (EB) and axon targeting to EB in the central brain^7,8^. Pdm3 is also important for the axon targeting of a type of tracheal dendrite (td) neurons^9^. In particular, td neurons that normally form synapse in the nerve cord change their target to the central brain by ectopic expression of Pdm3. Besides the neuronal functions of Pdm3, Pdm3 also acts as a repressor of abdominal pigmentation in *D. melanogaster*^10^, and plays a role in female-limited color dimorphism in abdomen of *D. montium*^11^. Despite these studies, it is still unknown how Pdm3 performs these neuronal and non-neuronal functions.

*pdm3*^*f00828*^ and *pdm3*^1^ homozygotes exhibit defects in axon targeting, odor perception, and locomotion^7,8^. *pdm3*^*f00828*^ allele has insertion of a piggyback element in an intron near the 3′ end of the *pdm3* gene, and *pdm3*^1^ has a premature stop codon in the middle of the coding region that results in the deletion of the POU domain^8^. We identified a new *pdm3* allele, *pdm3*^*m7*^, as a suppressor of lethality induced by Sol narae (Sona) overexpression in a genetic screen. Sona is a fly ADAMTS (A disintegrin and metalloprotease with thrombospondin motif) whose family members are secreted metalloproteases important for cell proliferation, cell survival and development^12–14^. We have shown that Sona positively regulates Wingless (Wg) signaling and is essential for fly development, cell survival, and Wg processing^12–14^. Wg is a prototype of Wnt family that initiates signal transduction cascade as extracellular signaling proteins, and activation of Wnt signaling leads to transcriptional induction of multiple genes for regulation of cell proliferation, cell survival, cell fate decision, and cell migration^15–17^. Wg is important for the development of all appendages, and the wing imaginal disc has been a great tool to study Wg signaling because Wg secreted from its dorsal-ventral midline is crucial for growth and development of wings^18^.

Wg also plays an essential role in the development of NMJ. During larval development, NMJs continue to form synaptic boutons that are specialized structures with axon terminals of motor neurons surrounded by reticular subsynaptical reticulum (SSR) formed by the plasma membrane of postsynaptic muscle^19^. Among multiple types of boutons such as type Ib, Is, II, and III, Wg is secreted at a high level from the glutamatergic type Ib bouton known as the main localization site of Wg protein and Wg signaling components, and is absent or at very low levels in other types of boutons^20^. Type Ib boutons also have more extensive SSR compared to other bouton types, so are easily detected by the high level of Discs-Large (Dlg) as a postsynaptic marker. Type Ib boutons in NMJs of *wg* mutants show reduction in bouton number but increase in bouton size^20–24^. Components in Wg signaling such as Arrow (Arr) that positively regulates Wg signaling as a coreceptor of Wg also shows its mutant phenotype similar to *wg*, but Shaggy (Sgg)/GSK3β that negatively regulates Wg signaling as a kinase shows opposite phenotype to *wg*^22,25^. Thus, dynamic regulation of Wg signaling is essential for the development of NMJ.

Secreted Wg also signals to the presynaptic motor neuron to regulate Futsch, one of the microtubule-associated proteins (MAPs)^26^. Futsch is a homolog of mammalian MAP1B, and both Futsch and MAP1B are phosphorylated at a conserved site by Sgg/GSK3β^27^. The phosphorylated MAP1B does not bind microtubules, which results in reduced stability of microtubules^3,28^. Therefore, localization of Futsch at NMJ faithfully reflects the stability of microtubules that is dynamically regulated by Wg signaling. Loss of *futsch* phenotype is similar to the loss of *wg* phenotype in NMJ^26^.

We report here that *pdm3* is identified as a suppressor of Sona-induced lethality. Based on the involvement of Sona in Wg signaling and the neuronal role of Pdm3, we specifically studied the roles of Pdm3 in NMJ. Similar to loss of *wg*, loss of *pdm3* in NMJ caused decrease in number but increase in size of boutons. Lethality induced by overexpressed Pdm3 was completely rescued by the knockdown of *wg* in motor neurons but not vice versa. This indicated that Pdm3 functions upstream to Wg, and prompted us to test whether Pdm3 can induce *wg* transcription. Indeed, transient expression of Pdm3 in wing discs induced *wg* transcription and Wg effector proteins. Based on these data, we propose that one of the main functions of Pdm3 in NMJ is to induce *wg* transcription.

## Results

### *pdm3* and *sona* have a positive genetic interaction

**Figure 1:**
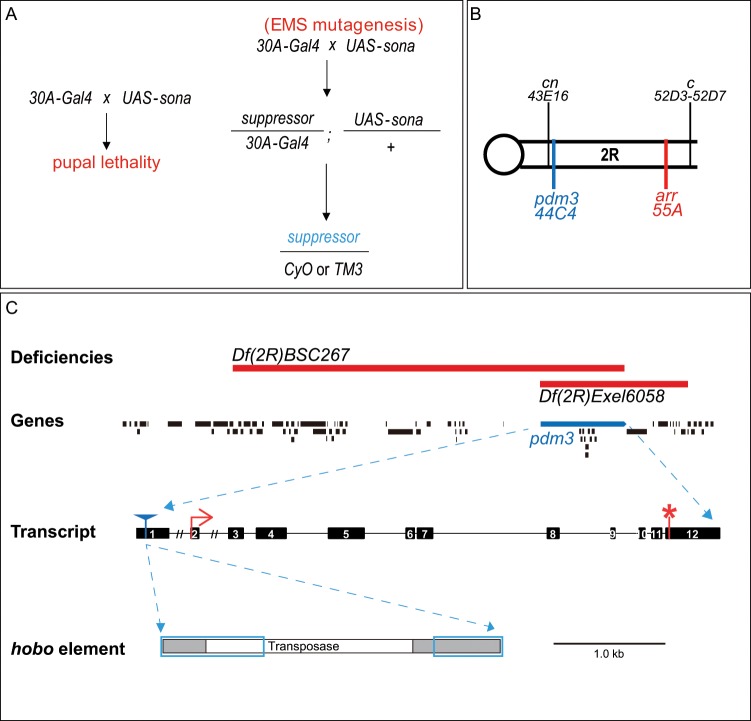
A lethality-based genetic screen for *sona* suppressors and characterization of the *m7* suppressor. (**A**) Scheme of a genetic screen for identifying suppressors that survive against late pupal lethality induced by Sona overexpression. EMS was used as a mutagen, and obtained suppressors from the screen were crossed with second and third chromosome balancers before further testing. (**B**) The *m7* suppressor was mapped by meiotic mapping, deficiency mapping, and complementation test. Multiple morphological markers are present in the second chromosome of a mapping line BDRC 4347, and the two markers, *cinnabar* (*cn*) as an eye color mutation and *curved* (*c*) as a wing shape mutation were identified as sites closely located to the two independent lethal sites of *m7* suppressor. Two lethal sites were separated by recombination with *CS* and subsequent complementation test with *pdm3*^*f00828*^ and *arr*^*2*^ identified that *m7* has two lethal mutations in *pdm3* and *arr* genes. (**C**) Two deficiency lines used for mapping are shown with deleted regions in red. Transheterozyogotes obtained by crossing the two deficiency lines do not have the *pdm3* gene. *pdm3*^*m7*^ has a defective *hobo* element inserted in an exon that represents the 5′ untranslated region. The blue boxes indicate remaining parts of the inserted *hobo* element. A red arrow marks the initiation codon and a red asterisk marks the termination codon. The scale bar is for the *hobo* element only.

As a first step toward understanding the function of *sona*, we carried out a lethality-based genetic screen using ethyl methanesulfonate (EMS) as a mutagen based on the late-pupal lethality induced by Sona driven by *30A-Gal4* (Fig. 1A). 89 rare survivors were obtained among 18,000 progenies from the cross between EMS-treated *30A-Gal4* males and untreated *UAS-sona* females. These survivors were balanced with *Sco/CyO* and *D/TM6* for the establishment of suppressor lines whose mutations are in the second and third chromosomes, respectively. Established lines were retested for the suppression of Sona-induced lethality, and 28 suppressors were maintained for further analysis (Fig. 1A). All suppressors showed lethality, and a few suppressors produced rare homozygous adults.

To map the position of the lethal site in suppressor *m7*, meiotic and deficiency mappings as well as complementation analysis were performed. Meiotic mapping was carried out by crossing the *m7* suppressor with a mapping line (BDRC #4347) that contains multiple morphological markers. The meiotic mapping revealed that the lethal region in *m7* is located in between the *cinnabar* (*cn*) and *curved* (*c*) (Fig. 1B). Subsequent deficiency mapping identified two different regions that are responsible for lethality, one near *cn* and the other near *c*. Complementation analysis then showed that *m7* has two independent mutations in *pdm3* and *arrow* (*arr*) genes on the right arm of the second chromosome (Fig. 1B). Pdm3 is a class VI POU domain transcription factor^5^, and Arr is a co-receptor of Wg ligand and essential for transduction of canonical Wg signaling^29^. The *m7* suppressor was crossed with *Canton-S* (*CS*) and their progeny was checked by complementation test with *pdm3*^*f00828*^ and *arr*^2^ mutants in order to find flies with a single mutation, *pdm3*^*m7*^ and *arr*^*m7*^.

Genomic sequencing revealed that *pdm3*^*m7*^ has a defective *hobo* element in the first exon of the *pdm3* gene that is upstream of the initiation codon (Fig. 1C) while *arr*^*m7*^ has a point mutation in the *arr* gene (in preparation). None of the other suppressors had the *hobo* element in the *pdm3* gene, indicating that insertion of the *hobo* element is unique to the *m7* suppressor, and occurred subsequent to the point mutation in the *arr* gene. We found that the level of Pdm3 is extremely low in *pdm3*^*m7*^ wing discs, establishing that insertion of the *hobo* element negatively affects the expression of Pdm3 (Fig. S1). Transheterozygotes of the two available deficiencies, *Df(2 R)BSC267* and *Df(2 R)Exel6058*, were missing only the *pdm3* gene in the entire genome, so *Df(2 R)BSC267*/*Df(2 R)Exel6058* flies were used as a deletion mutant of *pdm3* in this study (Fig. 1C).

We found that not only *pdm3*^*m7*^ but also *pdm3*^*f00828*^, *pdm3*^1^ and *pdm3 RNAi* driven by *30A-Gal4* completely suppressed the Sona-induced pupal lethality (n > 200 each). Thus, *pdm3*^*m7*^ is an authentic *sona* suppressor, and *pdm3* shows a positive genetic interaction with *sona*.

### Boutons of *pdm3* NMJs are decreased in number but increased in size, similar to *wg* NMJs

Further analysis of *sona* suppressors revealed that *sona* itself and most suppressors are linked to Wg signaling^12,13^, which raised an interesting possibility that Pdm3 is also involved in Wg signaling. We noticed that *pdm3* mutant larvae are slow in locomotion (Movie 1), implying a potential role of *pdm3* in NMJ. To address the relationship between *pdm3* and *wg* in NMJ, we stained *pdm3*^1^ and *wg*^*ts*^/*wg*^*CX4*^ NMJs of the late 3rd instar larvae for a presynaptic marker Horseradish peroxidase (HRP) and a postsynaptic marker Dlg to detect Type Ib boutons at muscles 6 and 7 in the 2^nd^ abdominal (A2) and the 3^rd^ abdominal (A3) segments^20^.

**Figure 2:**
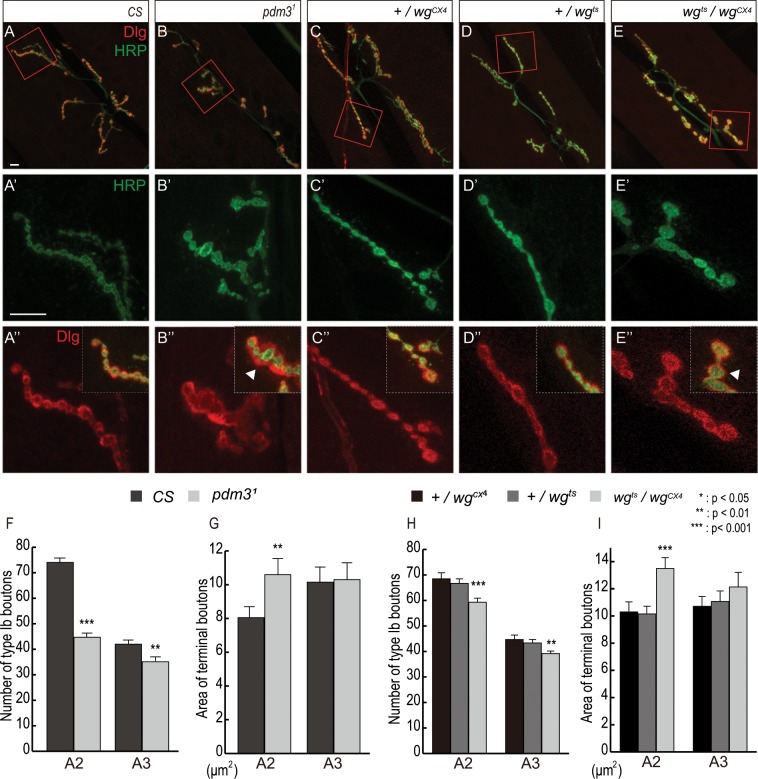
Boutons in *pdm3*^1^ and *wg*^*ts*^*/wg*^*cx4*^ mutant NMJs are decreased in number but increased in size. *CS* and *pdm3*^1^ were cultured at 25 ^o^C whereas *wg* mutants were cultured at 18 ^o^C in all figures. Type Ib boutons of NMJs at muscles 6/7 were stained for HRP (green) and Dlg^11^. Boxed regions in (**A**–**E**) are magnified in (**A’–E”**). (**A**–**E**) Boutons of NMJs in the A2 segment of control (**A**) and *pdm3*^1^. (**B**). Boutons of NMJs in the A2 segment of heterozygous controls (**C**,**D**) and *wg*^*ts*^*/wg*^*CX4*^. (**E**) Shorter and thicker branches and loosely organized boutons of *pdm3*^1^ and *wg*^*ts*^*/wg*^*CX4*^ are marked with arrowheads (**B”,E”**). (**F**–**I**) Number (**F**) and size (**G**) of terminal boutons of NMJs in A2 and A3 of wild-type and *pdm3*^1^. Number (**H**) and Size (**I**) of terminal boutons of NMJs in A2 and A3 of controls and *wg*^*ts*^*/wg*^*CX4*^. n = 51 for A2 and 37 for A3 of *CS*, 37 for A2 and 40 for A3 of *pdm3*^1^. n = 21 for A2 and 19 for A3 of +*/wg*^*CX4*^, n = 30 for A2 and 25 for A3 of +*/wg*^*ts*^, n = 44 for A2 and 48 for A3 of *wg*^*ts*^*/wg*^*CX4*^, n = 52 for A2 and 49 for A3 of +*/wg*^*CX4*^, and n = 50 for A2 and A3 of +*/wg*^*ts*^ and *wg*^*ts*^*/wg*^*CX4*^. *Represents p < 0.05; ** represents p < 0.01; *** represents p < 0.0001. Data are presented as mean ± SEM. Scale bars: 10 µm.

We found that number of boutons in *pdm3*^1^ NMJ was reduced by 40% and 16% compared to the wild-type counterparts in A2 and A3 segments, respectively (Fig. 2A,B,F). Bouton numbers in *pdm3*^*f00828*^ NMJ were reduced by 27% in A2 segment and those in *pdm3*^*m7*^ NMJ were only mildly reduced (Fig. S2; data not shown), so we focused our analysis on *pdm3*^1^ NMJ that shows the most pronounced phenotype. Consistent with the previous report, number of boutons in *wg*^*ts*^/*wg*^*CX4*^ NMJ was reduced by 12% and 11% in A2 and A3 segments, respectively (Fig. 2C–E,H).

We then carried out quantitative analysis on size of boutons in *pdm3* and *wg* NMJs. To measure the size of boutons, serial images of boutons were taken, the images were combined, and then area of the most distal bouton in the combined image was measured (Fig. 2G,I). Size of *pdm3*^1^ distal boutons was increased by 30% at A2 but was not increased at A3 segments compared to wild-type (Fig. 2G). Size of *wg*^*ts*^/*wg*^*CX4*^ boutons was increased by 32% and 11% in A2 and A3 segments, respectively, compared to the heterozygous controls, +*/wg*^*CX4*^ and +*/wg*^*ts*^ (Fig. 2I). This is in line with a previous report that *wg*^*ts*^ boutons are noticeably larger^20^.

Some *pdm3*^1^ boutons were not clearly separated from neighboring boutons (Fig. 2B”), which is also reported in *wg* boutons^20^. We defined the axon branch in which more than 50% of boutons are unseparated as ‘fused’ branch while those in which less than 50% of boutons are fused as ‘normal’ branch. We found that 30.5% of axon branches in A2 segments of *pdm3*^1^ NMJs are fused (36 out of 118). Only 3% of wild-type axon branches was fused based on this definition (8 out of 282).

One unique phenotype of *pdm3* boutons was an abnormally high level of Dlg in 42.9% of NMJs examined (33 out of 77, Fig. S3). Neither wild-type NMJs (0 out of 88) nor *wg* NMJs (0 out of 92) had high level of Dlg. This suggests that SSR is not properly developed in *pdm3*^1^ NMJs. We also checked the localized pattern of a glutamate receptor GluRIIA in *pdm3* and *wg* boutons. It has been shown that the GluRIIA pattern in wild-type bouton is cluster-like but that in *wg* boutons is diffused without any clusters^20^. Consistent with this report, cluster-like pattern of GluRIIA was found in *CS*, +/*wg*^*CX4*^, and +/*wg*^*ts*^ control boutons but *wg*^*ts*^*/wg*^*CX4*^ boutons showed diffused pattern (Fig. S4C–E). Unlike *wg* NMJs, the pattern of GluRIIA in *pdm3*1 NMJs was not noticeably different from control NMJs (Fig. S4A,B). Taken together, the loss of *pdm3* or *wg* phenotype decreased number but increased size of boutons, but *pdm3* and *wg* NMJs were dissimilar in the level of Dlg and the pattern of GluRIIA.

### Number of axon branches in *pdm3*^1^ NMJ is reduced

**Figure 3:**
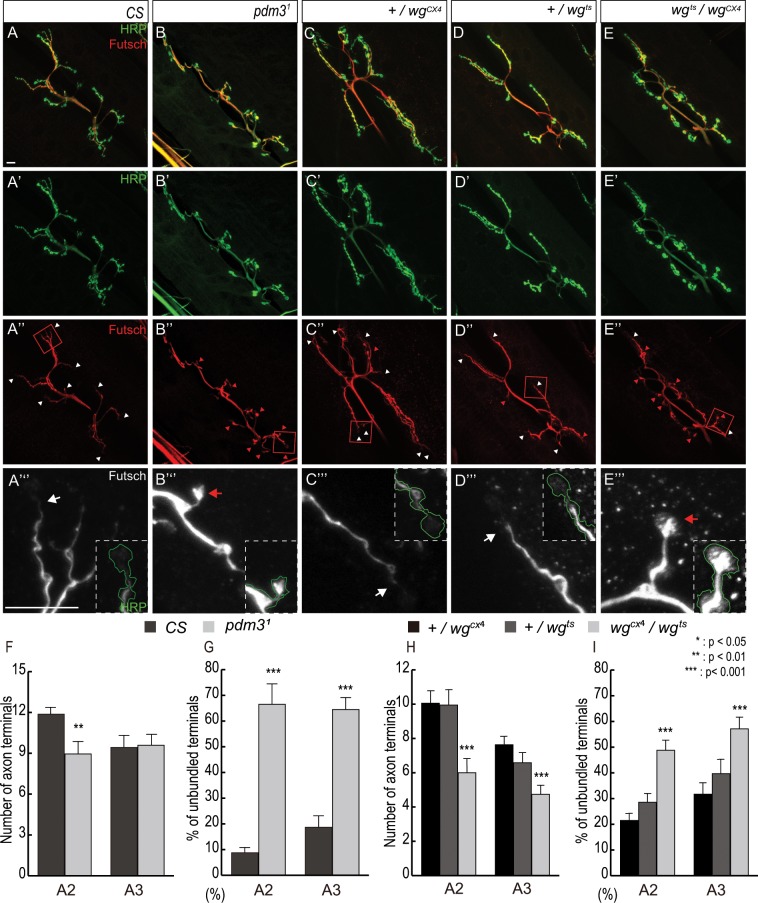
Decreased number of axon terminals and unstable microtubules in *pdm3*^1^ and *wg*^*ts*^*/wg*^*CX4*^ NMJs. The boutons of NMJs in muscles 6/7 at the A2 were stained for HRP (green) and Futsch (white). The boxed regions in (A’–E’) are magnified in (A”–E”). The white arrowhead and arrows indicate bundled Futsch-positive terminals, and red arrowheads and arrows indicate unbundled Futsch-positive terminals. Number of Futsch-positive terminals represents the number of branches in NMJs. (**A,B**) NMJs of the control and *pdm3*^1^ stained for HRP (**A**,**B**) and Futsch (**A’,B’**). (**C**–**E**) NMJs of the +*/wg*^*CX4*^ (**C**), +*/wg*^*ts*^ (**D**) and *wg*^*ts*^*/wg*^*CX4*^ (**E**) stained for HRP. (**F,G**) Number of Futsch terminals (**F**) and percentage of unbundled axon terminals (**G**) in the type Ib boutons of control and *pdm3*^1^. (**H,I**) Number of Futsch-positive terminals (**H**) and percentage of unbundled axon terminals (**I**) in the type Ib boutons of +*/wg*^*CX4*^, +*/wg*^*ts*^, and *wg*^*ts*^*/wg*^*CX4*^. n = 51 for A2 and 37 for A3 of *CS*, 37 for A2 and 40 for A3 of *pdm3*^1.^ n = 21 for A2 and 19 for A3 of +/*wg*^*CX4*^, n = 30 for A2 and 25 for A3 of +/*wg*^*ts*^, 44 for A2 and 48 for A3 of *wg*^*ts*^*/wg*^*CX4*^. *Represents p < 0.05; ** represents p < 0.01; *** represents p < 0.0001. Data are presented as mean ± SEM. Scale bars: 10 µm.

Wg signaling is required for the formation of new branches from an existing exon, and these new branches can be visualized by Futsch^20,22^. We found that number of axon branches in *pdm3*^1^ NMJs was decreased by 25% at the A2 segment and was unchanged at the A3 segment (Fig. 3A”,B”,F). *wg*^*ts*^*/wg*^*CX4*^ NMJs showed 40% and 33% reduction in number of axon branches at the A2 and A3 segments, respectively (Fig. 3C”–E”,H). Therefore, Pdm3 is important in A2 and Wg is important for both A2 and A3 for the formation of exon branches.

Stable microtubule-bound Futsch appears as a filamentous bundle that pass through the center of NMJ axon^20,22,26^. Interestingly, the distal bouton at the end of each axon branch visualized by Futsch shows four distinct shapes: a bundled shape and three types of unbundled shapes such as looped, splayed, and diffused/punctate^20^. Splayed or diffused/punctate axon terminals indicate that microtubules are unstable due to transition to new axonal growth, while looped axon terminals indicate paused growth cones^20,30^. Proportion of distal boutons with unbundled shape is increased by mutations that affect NMJ expansion such as *wg* and *futsch*^20,26^. Magnified images of wild-type NMJs showed that less than 10% and 20% of the axon terminals at A2 and A3 are unbundled, respectively (Fig. 3A”,A”’,G). In contrast, number of unbundled terminals was increased 7.3 and 3.4 times in A2 and A3 segments of *pdm3*^1^ NMJs compared to wild-type, respectively (Fig. 3B”,B”’,G; red arrow and arrowheads). All unbundled axon terminals in *pdm3* NMJs were either splayed or diffused/punctate, and looped axon terminals were not detected. Number of splayed or diffused/punctate terminals in *wg* NMJs was also increased about two times in A2 and A3 segments compared to wild-type (Fig. 3I). Thus, proportion of splayed or diffused/punctate terminals in A2 and A3 segments was significantly increased in both *pdm3* and *wg* NMJs.

Increase in number of splayed or diffused/punctate terminals in *pdm3* NMJs suggests that microtubules in *pdm3* NMJs are unstable. To directly address this point, we visualized axon terminals with α-Tubulin and Futsch. In fact, signals from α-Tubulin and Futsch staining were much weaker in axon branches of *pdm3* NMJ compared to wild-type (Fig. S5A,B). In case of *wg* NMJs, signal from α-Tubulin staining was substantially reduced in entire axon compared to wild-type (Fig. S5C–E). In summary, microtubules become unstable, which may lead to reduced number of axon branches in both *pdm3* and *wg* NMJs.

### Pdm3 expression in motor neuron is important for NMJ growth

**Figure 4:**
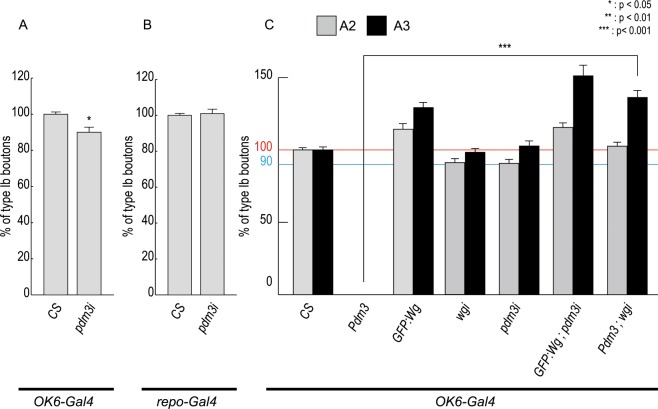
Pdm3 is required in motor neurons and acts upstream to Wg. (**A,B**) The number of boutons in *OK6-Gal4*/+ as a control (**A**) or in *repo-Gal4/*+ as a control (**B**) is set at 100% and those in *OK6* > *pdm3i* were divided by the control bouton number and multiplied by 100 for bar graphs. n = 27 for control, 32 for *OK6* > *wgi*, and 18 for *OK6* > *pdm3i*. n = 23 for control, 21 for *repo* > *wgi*, and 34 for *repo* > *pdm3i*. (**C**) The number of boutons in the A2 and A3 segments when *GFP-wg*, *wgi*, *pdm3*, *pdm3i* are singly expressed or coexpressed by *OK6-Gal4*. n = 27 for A2 and 34 for A3 of *CS*, 18 for A2 and 17 for A3 of *GFP-wg*, 32 for A2 and 30 for A3 of *wgi*, 18 for A2 and 20 for A3 of *pdm3i*, 22 for A2 and A3 of *GFP-wg*;*pdm3i*, 22 for A2 and 20 for A3 of *GFP-wgi*;*pdm3*. *Represents p < 0.01; ** represents p < 0.0001. Data are presented as mean ± SEM.

Wg secreted from motor neuron and glia is important for growth and differentiation of presynaptic terminals^20,24^. To figure out which cell type among motor neuron and glia expresses Pdm3, we expressed *pdm3 RNAi* in motor neurons by the *OK6-Gal4* driver and in glia by the *repo-Gal4* driver (Fig. 4A,B). These two *Gal4* lines have been used to show cell specificity of a given protein in numerous reports^22,24,31–34^. Knockdown of *pdm3* by *OK6-Gal4* caused 10% reduction in bouton number, suggesting that *pdm3* is required in neurons for NMJ growth (Fig. 4A). Knockdown of *pdm3* by *repo-Gal4* did not change bouton number, suggesting that Pdm3 expression in glia is not required for NMJ growth (Fig. 4B).

We then asked whether expression of Pdm3 by *OK6-Gal4* rescues *pdm3*^1^ NMJ phenotype. To this end, we generated *UAS-pdm3 pdm3*^1^*/CyO-GFP* and *OK6-Gal4 pdm3*^1^*/CyO-GFP* flies and checked the phenotype of their progeny, *UAS-pdm3 pdm3*^1^/*OK6-Gal4 pdm3*^*1*^. Unexpectedly, *pdm3*^1^ homozygotes were cold sensitive and could not grow at the temperature lower than 22 °C, but Pdm3 overexpression by *OK6-Gal4* induced lethality at the temperature higher than 24 °C. Due to this temperature restraint, *UAS-pdm3 pdm3*^1^/*OK6-Gal4 pdm3*^1^ larvae were obtained only at 23 °C at a very low frequency. NMJs of these larvae showed increase in bouton number, and decrease in bouton size, and normalized level of Dlg compared to *pdm3*^1^ NMJs (Fig. S6). Taken together, overexpressed Pdm3 in motor neurons rescued the loss of *pdm3* phenotype in NMJs.

Our results so far have shown that bouton number and size of *pdm3* NMJs are more severely affected in A2 than A3, so we examined the expression pattern of Pdm3 in ventral ganglion where cell bodies of motor neurons are present in order to examine the level of Pdm3 along the anterior-posterior (AP) axis. We found that Pdm3 is expressed more in the anterior part than posterior part of ventral ganglion, which is consistent with severer *pdm3* phenotype in A2 than A3 (Fig. S7). Further analysis with more refined markers will help understand the effect of this AP gradient of Pdm3 on NMJ growth.

### Pdm3 acts upstream to Wg in neurons

Similarity between *pdm3* and *wg* NMJs prompted us to examine the genetic relationship between *pdm3* and *wg* by co-expression of the two among *pdm3, GFP-wg, pdm3 RNAi*, and *wg RNAi* (Fig. 4C). As controls, bouton number of NMJs in these *UAS* lines was counted, which turned out to be similar to that of *CS* (Fig. S8). When we overexpressed *pdm3* or *wg* by *OK6-Gal4*, *pdm3* caused larval lethality, and *wg* increased bouton numbers in both A2 and A3 segments (Fig. 4C). When we knocked down *pdm3* or *wg* by the same *Gal4*, the bouton number of the A2 segment was reduced by 10%, but that of the A3 segment was not changed in both cases. When *GFP-wg* and *pdm3 RNAi* were co-expressed, increase in the bouton number by GFP-Wg was not affected by *pdm3i* (Fig. 4C). When *pdm3* and *wg RNAi* were co-expressed, however, lethal phenotype by overexpressed *pdm3* was completely rescued by knockdown of *wg*. Therefore, *wg* is epistatically downstream to *pdm3*. This result raised an interesting possibility that Pdm3 may regulate *wg* transcription.

### *pdm3* adults exhibit defects in locomotion, planar cell polarity and wing posture

**Figure 5:**
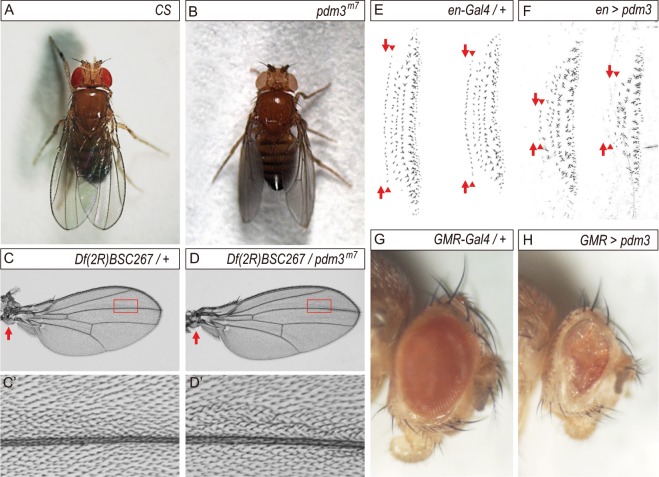
Loss or gain of *pdm3* phenotypes in wings, denticles and eyes. (**A,B**) Wing posture of *pdm3* homozygotes and transheterozygotes. *CS* flies have normal wings. (**A**) *pdm3*^*m7*^ flies have opaque drooping wings. Opaqueness is due to the lack of adhesion between the dorsal and ventral wing blades. (**C,D**) The +/*Df(2 R)BSC267* control wing (**C**) and the *pdm3*^*m7*^/*Df(2 R)BSC267* wing (**D**). PCP phenotype is observed along the distal half of the L3 vein, and the most affected region is marked with the red box and magnified in (**C’,D’**). There are no structural defects in the hinge (arrows in **C**,**D**). (**E,F**) Ventral denticle bands of A5 and A6 of the control *en-Gal4*/+ (**E**) and *en* > *pdm3* 1^st^ instar larvae. (**F**) Pdm3 overexpression by *en-Gal4* induces embryonic lethality and defects in ventral denticles. *en*-*Gal4* drives expression in the last row of naked cuticle (arrows) and the first row of denticle bands (arrowheads). (**G,H**) Adult eyes of the control *GMR-Gal4*/+ (**G**) and *GMR* > *pdm3* (**H**).

Pdm3 has both neuronal and non-neuronal roles in fly development (see Introduction). 100% of *pdm3*^*f00828*^*, pdm3*^*m7*^, and *pdm3*^1^ adults (n = ~50 each) had other defects such as wing drooping (Fig. 5A,B), planar cell polarity (PCP) phenotype in a posterior region near the L3 vein (Fig. 5C,D), and incomplete adhesion between dorsal and ventral blades of wings (Fig. S9, Table 1 in Supplementary Information). These phenotypes of *pdm3* mutants suggest that Pdm3 plays previously unidentified roles in wing development. Therefore, we decided to use wing discs to study the relationship between *pdm3* and *wg*.

Pdm3 was highly expressed in both proximal and distal hinge regions in and near where *patched* (*ptc*) is expressed (Fig. S10A,A’). This Pdm3 pattern is genuine because Pdm3 was not detected in the *ptc* region of *ptc* > *pdm3i* discs (Fig. S10B,B”). Expression of Pdm3 in the hinge region may be responsible for the wing drooping phenotype of *pdm3* mutants although there were no visible defects in adult wing hinges (Fig. 5C,D). No change in the level of Wg was observed in the *ptc* > *pdm3i* wing discs, suggesting that loss of *pdm3* does not affect *wg* transcription in the DV midline of the wing pouch region (Fig. S11).

### Transient expression of Pdm3 induces *wg* transcription

**Figure 6:**
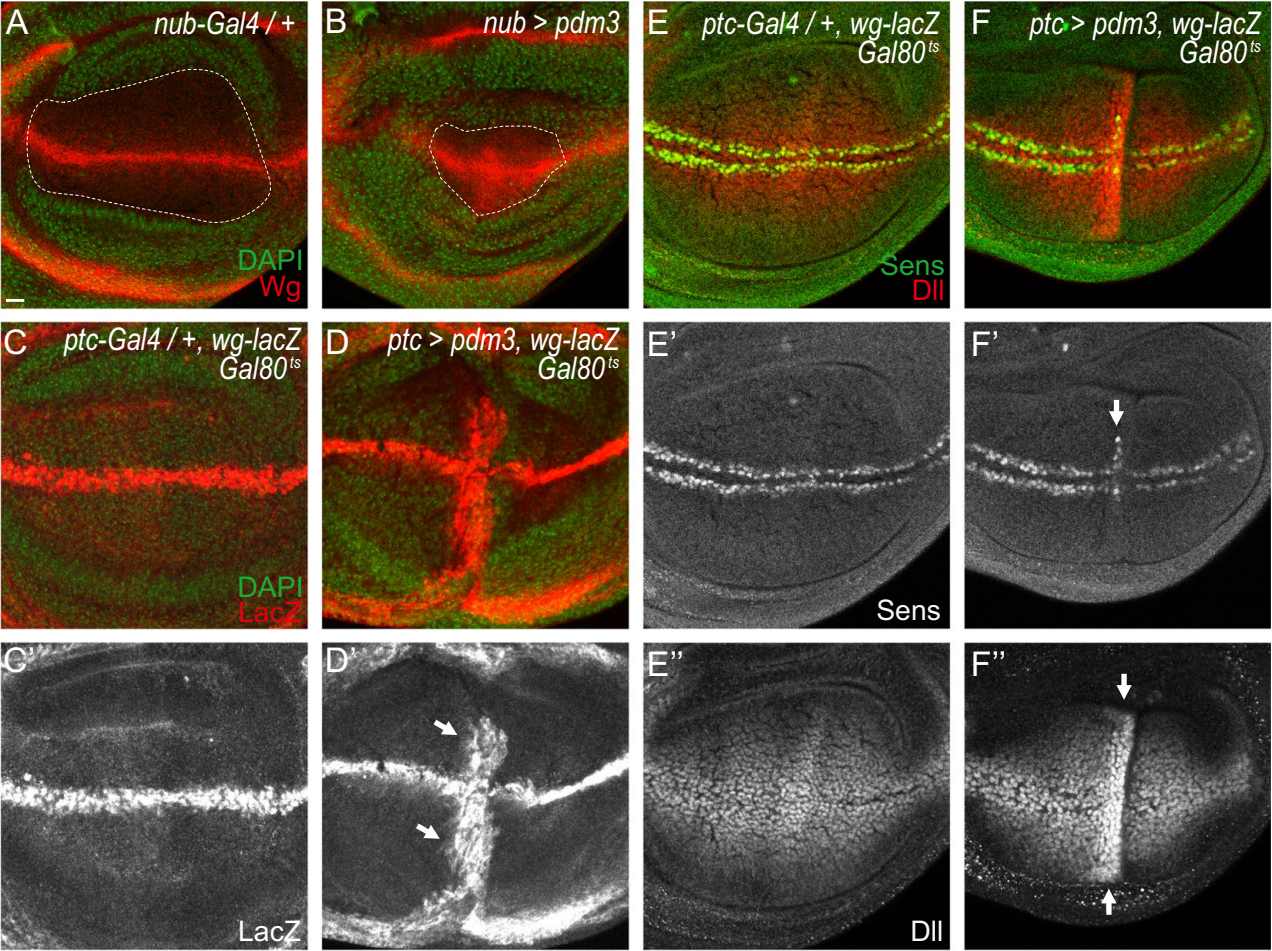
Pdm3 induces *wg* transcription. (**A,B**) *nub* > *pdm3* wing discs (**B**) have smaller wing pouch than control *nub-Gal4*/+ (**A**). (**C,D**) Induction of Wg-LacZ (arrows) after 36 hours of *pdm3* expression in *ptc* > *pdm3 Gal80*^*ts*^ discs (**D**) compared to the control discs (**C**). (**C’,D’**) are black and white images of (**C**,**D**). (**E,F**) Induction of Sens (**F’**) and Dll (**F”**) marked with arrows, compared to control (**E-E”**).

To understand the role of Pdm3 in relation with Wg, we carried out gain of function analyses using multiple *Gal4* lines. Overexpression of Pdm3 induced embryonic to pupal lethality with all *Gal4* lines used in this study (Table 2 in supplementary information). In case of *en-Gal4* driver that caused embryonic lethality, some rare larval escapers were shorter than controls and had abnormal denticle patterns in the ventral epidermis (Fig. 5E,F). Pdm3 overexpression by other tissue-specific *Gal4* lines also reduced size of affected tissues. For instance, Pdm3 expression by *GMR-Gal4* generated small eyes (Fig. 5G,H), and that by *nub-Gal4* caused mostly pupal lethality and loss of wings in rare adults (Table 2 in supplementary information). Consistent with this phenotype of *nub* > *pdm3* wings, size of all *nub* > *pdm3* wing discs examined was smaller than control wing discs (n = 14 each, Fig. 6A,B).

One interesting finding was increase in the level of Wg at the DV midline of *nub* > *pdm3* wing discs (Fig. 6A,B). To examine this phenomenon further, we transiently expressed *pdm3* with *Gal80*^*ts*^ system using *ptc-Gal4* for 6, 12, 24, 36 and 48 hours at 30 °C in order to avoid lethality by Pdm3 overexpression, and checked the level of wg-LacZ as a marker for *wg* transcription. We found that wg-LacZ was ectopically expressed in the *ptc* region after 36 or 48 hours but not before 36 hours of transient Pdm3 expression (Fig. 6C,D). The downstream effector proteins of Wg signaling, Distal-less (Dll) and Sensless (Sens), were also induced at the *ptc* region (Fig. 6E,F). Thus, *pdm3* directly or indirectly activates transcription of *wg*.

## Discussion

We report here that Pdm3 regulates growth and development of NMJs. *pdm3* mutants showed increase in bouton size and decrease in bouton number, which are similar to the phenotype of *wg* mutants. Lethality induced by the overexpression of Pdm3 was rescued by knockdown of *wg* in NMJ, indicating that Pdm3 functions upstream to Wg. Furthermore, overexpression of Pdm3 induced *wg* transcription in wing discs. We propose here that a major function of Pdm3 in motor neurons is to induce *wg* transcription, and secreted Wg from motor neurons regulates growth, development, and maturation of both pre- and post-synaptic regions of NMJ.

The mammalian homolog of Pdm3 is Brain-5 (Brn-5)/POU class 6 homeobox 1 (POU6F1) mainly expressed in brain and spinal cord. Brn-5 is heavily expressed in embryonic brain but also expressed in adult brain and multiple adult organs such as kidney, lung, testis, and anterior pituitary^35^. In developing brain, Brn-5 is expressed in postmitotic neurons after neuronal progenitor cells exit cell cycle in the early process of terminal neuronal differentiation^36^. Therefore, both Pdm3 and Brn-5 function in differentiation of neurons. Interestingly, ectopic expression of Brn-5 inhibits DNA synthesis^37^, which is similar to cell cycle arrest phenotype by Wg overexpression^38^. Given the homology between Pdm3 and Brn-5 as well as functional similarities, Brn-5 may also induce *wnt* transcription.

Most of Pdm3 functions identified so far are related to the maturation of neurons such as olfactory neurons, R neurons and td neurons as well as their postsynaptic partners^7–9^. Ectopic expression of Pdm3 induced lethality without exception, indicating that expression of Pdm3 in fly tissues is generally repressed *in vivo* in order to express Wg under the strict spatiotemporal control. An important question is whether Pdm3 directly transcribe *wg*. We found that *wg* transcription is induced only after 36 hours of transient overexpression of Pdm3. It is possible that the level of Pdm3 needs to be over a threshold to induce *wg* transcription. Alternatively, Pdm3 may need to turn on other components to indirectly induce *wg* transcription. DNA sequence of Brn-5 binding site has been reported^39–41^, so analysis on *wg* and *wnt* regulatory regions will help understand the mechanism of *wnt* induction by Pdm3 and Brn-5.

We consistently found more significant NMJ phenotypes in A2 than A3 in both *pdm3* and *wg* mutants. Therefore, *pdm3* and *wg* may play more prominent roles in the A2 than the A3 segment. In fact, the level of Pdm3 was higher in the anterior region than the posterior region of ventral ganglion, which suggests that more Wg may be present in the NMJs of anterior abdominal segments. Consistent with this idea, the number of type Ib boutons in the A2 segment was 1.8 times more than A3 segment. One difference between *pdm3* and *wg* mutants is the lack of certain phenotypes in the A3 segment of *pdm3* NMJs: the size of boutons and the number of axon terminals in A3 were not affected in *pdm3* mutant. It is possible that Pdm3 turns on both common and segment-specific genes besides *wg*, and A3 segment-specific components may alleviate the loss of *wg* phenotype in the A3 segment. Similarly, other proteins induced by Pdm3 may also play important roles in NMJ growth, differentiation and maintenance. In fact, multiple signaling pathways including Glass-bottom-boat (Gbb) pathway also play roles in NMJ development^42,43^. Gbb is secreted from muscles and induces development of both pre- and post-synaptic structures, similar to Wg signaling.

We identified a defective *hobo* element in the *pdm3*^*m7*^ allele. The *hobo* element belongs to Ac family found in maize and has short inverted terminal repeats^44^. Laboratory and wild strains of *D. melanogaster* have average 28 and 22 copies of *hobo* elements in the genome that are either full-length or defective, respectively^45,46^. Because other suppressors identified in the genetic screen using Sona overexpression did not have *hobo* element in the *pdm3* gene, the transposition of the *hobo* element to the *pdm3* gene may have occurred subsequent to the generation of a point mutation in the *arr* gene by EMS. Since both *arr* and *pdm3* are positively involved in Wg signaling, this *hobo* insertion may have helped the original *arr*^*m7*^ mutation to further decrease the activity of Wg signaling under the condition of Sona overexpression.

Besides the neuronal roles of Pdm3, all *pdm3* mutants show minor but consistent defects in planar cell polarity in a restricted region of the wing as well as adhesion between the dorsal and ventral wing blades. Other phenotypes such as wing drooping and premature death were also observed in all *pdm3* mutants, but these may be due to malformation of synaptic structures. Pdm3 also plays a role in female-limited color dimorphism in abdomen of *D. montium*^11^. The authors found in sexually dimorphic females that the first intron of the *pdm3* gene has four tandem sets with predicted binding sites for the HOX gene *Abdominal-B* (*Abd-B*) and the sex determination gene *doublesex* (*dsx*). Interestingly, it has been shown that Wg expression is repressed by the combinatory work of Abd-B and Dsx proteins^47^. Taken together, it is possible that transcription of *wg* and *pdm3* is co-repressed by Abd-B and Dsx. Such co-repression of *wg* and *pdm3* transcription may be also required for synaptic growth and differentiation in neurons. Further studies on Pdm3 will help understand how this understudied transcription factor is involved in the final differentiation of various cell types.

## Materials and Methods

### Fly strains

*Ok6-Gal4, BG57-Gal4* and *repo-Gal4*^48^ were obtained from S.-B. Lee’s lab, and *UAS-pdm3*^7,8^, *UAS-GFP:wg*^49^, *pdm3*^*f*^*°°*^828^ and *pdm3*^1^ ^7,8^ were obtained from the labs that produced them. *UAS-sona*^12^ is produced in our lab. *UAS-wg RNAi* (#4889R-3) was obtained from Fly Stocks of National Institute of Genetics. All other lines such as *UAS-GFP* (#1533), *UAS-GFP:CD8* (#5130), *UAS-pdm3* RNAi (#26749), *Df(*2* R)BSC267, DF(2 R)Exel6058*, were obtained from Bloomington Drosophila Stock Center.

### Immunohistochemistry

Immunohistochemical staining of larval wing discs was performed as described^12^, and that of NMJs was performed as described with slight modifications^50,51^. To obtain larvae for NMJ analysis, flies were cultured at 25 °C except *wg* mutants at 18 °C. To control the population size of larvae in each vial, eggs were harvested on grape plates and incubated at 25 °C for 24 hours. Then, 30 larvae were transferred to a food vial, and were cultured until the wandering larval stage for dissection. In case of *wg* larvae, 30 larvae were cultured at 18 °C until dissection. For NMJ staining, larvae were dissected with HL3.1 solution on sylgard plates and fixed for 20 minutes in 5% formaldehyde/HL3.1 solution^52^ or Bouin’s solution^50^. Fixed samples were rinsed 3 to 4 times with PBS or HL3.1 and then were blocked in 5% BSA in PBS before antibody treatment. The following antibodies were used: anti-Pdm3 (rat, 1:100)^7^, anti-β-Gal (chicken, 1:100; Abcam ab134435), anti-Dlg (rabbit, 1:500)^53^, anti-HRP-Cy3 (1:100; Jackson ImmunoResearch), anti-Wg (mouse, 1:1,000; DSHB 4D4), anti-Futsch (mouse, 50:1; DSHB 22C10), anti-α-Tubulin (mouse, 1:200; Sigma MAB1864), anti-Glutamate receptor IIA (mouse, 1:10; DSHB concentrated 8B4D2 (MH2B)). For GluRIIA staining, the samples were fixed by Bouin’s solution.

### Image capture and quantitative analysis of boutons

We stained the late 3rd instar larvae for a presynaptic marker HRP and a postsynaptic marker Dlg to detect NMJs. Type Ib boutons have more extensive SSR compared to other bouton types (Is, II, and III), so are easily detected by the high level of Dlg^19^. Therefore, type Ib boutons are defined as round-shaped structures in NMJ branches that are stained with HRP and have high level of Dlg, and only ones that were qualified to this definition were counted as boutons. To obtain images containing boutons, type Ib boutons visualized with HRP and Dlg were taken with 1 μm interval for 7–10 Z stacks at 400X magnification by a confocal laser microscope of Carl Zeiss (NFEC-2010-09-141569) with Zen 2009 program. To manually count number of boutons, all Z stack images were then merged and type Ib boutons at muscles 6 and 7 in A2 or A3 segments in a given image were counted. The number of images used for counting for each genotype was 17–52. To measure size of boutons, images of terminal boutons were captured at 2,000X magnifications and then area of boutons in merged images was measured by Zen 2009 program.

### Cuticle preparation of larvae

The cuticle preparation was performed as described with slight modifications^54^. Flies were put into a chamber with a grape juice-containing agar plate that has yeast paste at the center. After 4 hours of egg-laying, plates were incubated for 20 hours at 25 °C. Larvae were transferred to distilled water on cover glass, and washed again with distilled water. Water was then removed and the 1:1 mixture of lactic acid and Hoyer’s mount solution was applied. After waiting for about 1 minute, the sample on a cover glass was placed on the slide glass, and then incubated for overnight at 65 °C.

### Statistical analysis

Statistical analysis was performed using ANOVA to compare different genotypes to a wild-type control within experimental groups. Data are presented as mean ± SEM. To determine statistical significance, t-test and one-way ANOVA of Microsoft Excel 2019 were used.

## Supplementary information

Movie 1.

Supplementary Information.

## References

[1] Deis, G. *et al*. TPX superconducting tokamak magnet system - 1995 design and status overview. *Sofe ‘95 -* 1*6th Ieee/Npss Symposium on Fusion Engineering, Vols 1 and 2*, 1383–1388 (1995).

[2] Malik Vikas, Zimmer Dennis, Jauch Ralf (2018). Diversity among POU transcription factors in chromatin recognition and cell fate reprogramming. Cellular and Molecular Life Sciences.

[3] Gold DA, Gates RD, Jacobs DK (2014). The early expansion and evolutionary dynamics of POU class genes. Mol. Biol. evolution.

[4] Herr W (1988). The Pou Domain - a Large Conserved Region in the Mammalian Pit-1, Oct-1, Oct-2, and Caenorhabditis-Elegans Unc-86 Gene-Products. Gene Dev..

[5] Ryan AK, Rosenfeld MG (1997). POU domain family values: Flexibility, partnerships, and developmental codes. Gene Dev..

[6] Herr W, Cleary MA (1995). The Pou Domain - Versatility in Transcriptional Regulation by a Flexible 2-in-One DNA-Binding Domain. Gene Dev..

[7] Tichy AL, Ray A, Carlson JR (2008). A new Drosophila POU gene, pdm3, acts in odor receptor expression and axon targeting of olfactory neurons. Journal of Neuroscience.

[8] Chen CK, Chen WY, Chien CT (2012). The POU-domain protein Pdm3 regulates axonal targeting of R neurons in the Drosophila ellipsoid body. Dev. Neurobiol..

[9] Qian CS, Kaplow M, Lee JK, Grueber WB (2018). Diversity of Internal Sensory Neuron Axon Projection Patterns Is Controlled by the POU-Domain Protein Pdm3 in Drosophila Larvae. Journal of Neuroscience.

[10] Rogers WA (2014). A survey of the trans-regulatory landscape for Drosophila melanogaster abdominal pigmentation. Developmental Biol..

[11] Yassin A (2016). The pdm3 Locus Is a Hotspot for Recurrent Evolution of Female-Limited Color Dimorphism in Drosophila. Curr. Biol..

[12] Kim, G. W. *et al*. Sol narae (Sona) is a Drosophila ADAMTS involved in Wg signaling. *Sci Rep-Uk* **6**, 10.1038/srep31863 (2016). 10.1038/srep31863 PMC4989167 27535473

[13] Won, J. H. *et al*. ADAMTS Sol narae cleaves extracellular Wingless to generate a novel active form that regulates cell proliferation in Drosophila. *Cell Death & Disease* **10**, 10.1038/s41419-019-1794-8 (2019). 10.1038/s41419-019-1794-8 PMC6646336 31332194

[14] Tsogtbaatar, O. *et al*. An ADAMTS Sol narae is required for cell survival in Drosophila. *Sci Rep-Uk* **9**, 10.1038/s41598-018-37557-9 (2019). 10.1038/s41598-018-37557-9 PMC6362049 30718556

[15] Clevers H (2006). Wnt/beta-catenin signaling in development and disease. Cell.

[16] Amin N, Vincan E (2012). The Wnt signaling pathways and cell adhesion. Front. Biosci. (Landmark Ed.).

[17] Logan CY, Nusse R (2004). The Wnt signaling pathway in development and disease. Annual Review of Cell and Developmental Biology.

[18] Swarup S., Verheyen E. M. (2012). Wnt/Wingless Signaling in Drosophila. Cold Spring Harbor Perspectives in Biology.

[19] Menon KP, Carrillo RA, Zinn K (2013). Development and plasticity of the Drosophila larval neuromuscular junction. Wires Dev. Biol..

[20] Packard M (2002). The drosophila wnt, wingless, provides an essential signal for pre- and postsynaptic differentiation. Cell.

[21] Mathew D (2005). Wingless signaling at synapses is through cleavage and nuclear import of receptor DFrizzled2. Science.

[22] Miech C, Pauer HU, He X, Schwarz TL (2008). Presynaptic Local Signaling by a Canonical Wingless Pathway Regulates Development of the Drosophila Neuromuscular Junction. Journal of Neuroscience.

[23] Ataman B (2008). Rapid activity-dependent modifications in synaptic structure and function require bidirectional Wnt signaling. Neuron.

[24] Kerr KS (2014). Glial Wingless/Wnt Regulates Glutamate Receptor Clustering and Synaptic Physiology at the Drosophila Neuromuscular Junction. Journal of Neuroscience.

[25] Franco B (2004). Shaggy, the homolog of glycogen synthase kinase 3, controls neuromuscular junction growth in Drosophila. Journal of Neuroscience.

[26] Roos J, Hummel T, Ng N, Klambt C, Davis GW (2000). Drosophila Futsch regulates synaptic microtubule organization and is necessary for synaptic growth. Neuron.

[27] Gogel S, Wakefield S, Tear G, Klambt C, Gordon-Weeks PR (2006). The Drosophila microtubule associated protein Futsch is phosphorylated by Shaggy/Zeste-white 3 at an homologous GSK3 beta phosphorylation site in MAP1B. Mol. Cell Neurosci..

[28] Ciani L, Krylova O, Smalley MJ, Dale TC, Salinas PC (2004). A divergent canonical WNT-signaling pathway regulates microtubule dynamics: Dishevelled signals locally to stabilize microtubules. Journal of Cell Biology.

[29] Wehrli M (2000). Arrow encodes an LDL-receptor-related protein essential for Wingless signalling. Nature.

[30] Dent EW, Callaway JL, Szebenyi G, Baas PW, Kalil K (1999). Reorganization and movement of microtubules in axonal growth cones and developing interstitial branches. Journal of Neuroscience.

[31] Freeman Marc R. (2015). DrosophilaCentral Nervous System Glia. Cold Spring Harbor Perspectives in Biology.

[32] Casas-Tintó Sergio, Arnés Mercedes, Ferrús Alberto (2017). Drosophila enhancer-Gal4 lines show ectopic expression during development. Royal Society Open Science.

[33] Sanyal S (2009). Genomic mapping and expression patterns of C380, OK6 and D42 enhancer trap lines in the larval nervous system of Drosophila. Gene Expr. Patterns.

[34] De Gregorio C, Delgado R, Ibacache A, Sierralta J, Couve A (2017). Drosophila Atlastin in motor neurons is required for locomotion and presynaptic function. Journal of Cell Science.

[35] Andersen B (1993). Brn-5 Is a Divergent Pou Domain Factor Highly Expressed in Layer-Iv of the Neocortex. Journal of Biological Chemistry.

[36] Cui, H. & Bulleit, R. F. Expression of the POU transcription factor Brn-5 is an early event in the terminal differentiation of CNS neurons. *J Neurosci Res* **52**, 625–632, doi:10.1002/(SICI)1097-4547(19980615)52:6<625::AID-JNR1>3.0.CO;2-A (1998). 10.1002/(SICI)1097-4547(19980615)52:6<625::AID-JNR1>3.0.CO;2-A 9669311

[37] Cui H, Bulleit RF (1997). Expression of the POU transcription factor Brn-5 inhibits proliferation of NG108-15 cells. Biochemical and Biophysical Research Communications.

[38] Johnston LA, Edgar BA (1998). Wingless and Notch regulate cell-cycle arrest in the developing Drosophila wing. Nature.

[39] Pereira JH, Kim SH (2009). Structure of human Brn-5 transcription factor in complex with CRH gene promoter. Journal of Structural Biology.

[40] Pereira JH, Ha SC, Kim SH (2008). Crystallization and preliminary X-ray analysis of human Brn-5 transcription factor in complex with DNA. Acta Crystallographica Section F: Structural Biology and Crystallization Communications.

[41] Andersen B (1993). Brn-5 is a divergent POU domain factor highly expressed in layer IV of the neocortex. Journal of Biological Chemistry.

[42] McCabe BD (2003). The BMP homolog Gbb provides a retrograde signal that regulates synaptic growth at the Drosophila neuromuscular junction. Neuron.

[43] Bayat V, Jaiswal M, Bellen HJ (2011). The BMP signaling pathway at the Drosophila neuromuscular junction and its links to neurodegenerative diseases. Current Opinion in Neurobiology.

[44] Calvi BR, Hong TJ, Findley SD, Gelbart WM (1991). Evidence for a Common Evolutionary Origin of Inverted Repeat Transposons in Drosophila and Plants - Hobo, Activator, and Tam3. Cell.

[45] Biemont C, Gautier C (1988). Localization Polymorphism of Mdg-1, Copia, I-Mobile-Element and P-Mobile-Element in Genomes of Drosophila-Melanogaster, from Data of Inbred Lines. Heredity.

[46] Ruiz MT, Carareto CMA (2003). Characterization of hobo element copy number and integrity in Brasilian populations of Drosophila melanogaster. Hereditas.

[47] Wang W, Kidd BJ, Carroll SB, Yoder JH (2011). Sexually dimorphic regulation of the Wingless morphogen controls sex-specific segment number in Drosophila. Proc. Natl Acad. Sci. U S Am..

[48] Nahm M (2013). Spartin regulates synaptic growth and neuronal survival by inhibiting BMP-mediated microtubule stabilization. Neuron.

[49] Pfeiffer S, Ricardo S, Manneville JB, Alexandre C, Vincent JP (2002). Producing cells retain and recycle Wingless in Drosophila embryos. Curr. Biol..

[50] Budnik V, Gorczyca M, Prokop A (2006). Selected methods for the anatomical study of Drosophila embryonic and larval neuromuscular junctions. Int. Rev. Neurobiol..

[51] Brent JR, Werner KM, McCabe BD (2009). Drosophila larval NMJ dissection. Journal of Visualized Experiments: JoVE.

[52] Stewart BA, Atwood HL, Renger JJ, Wang J, Wu CF (1994). Improved stability of Drosophila larval neuromuscular preparations in haemolymph-like physiological solutions. J. Comp. Physiol. A.

[53] Cho KO, Chern J, Izaddoost S, Choi KW (2000). Novel signaling from the peripodial membrane is essential for eye disc patterning in Drosophila. Cell.

[54] Alexandre C (2008). Cuticle preparation of Drosophila embryos and larvae. Methods Mol. Biol..

