# Supplementary material for: POU domain motif3 (Pdm3) induces wingless (wg) transcription and is essential for development of larval neuromuscular junctions in Drosophila
Source: Sci Rep. 2020 Jan 16;10:517. doi: 10.1038/s41598-020-57425-9 (PMC6965103; doi:10.1038/s41598-020-57425-9)
Supplement: Supplementary file 2 — Supplementary Information. [file 41598_2020_57425_MOESM2_ESM.pdf]

POU domain motif3 (Pdm3) induces *wingless* (*wg*) transcription and is essential for development of larval neuromuscular junctions in *Drosophila*

Yeon Kim and Kyung-Ok Cho*

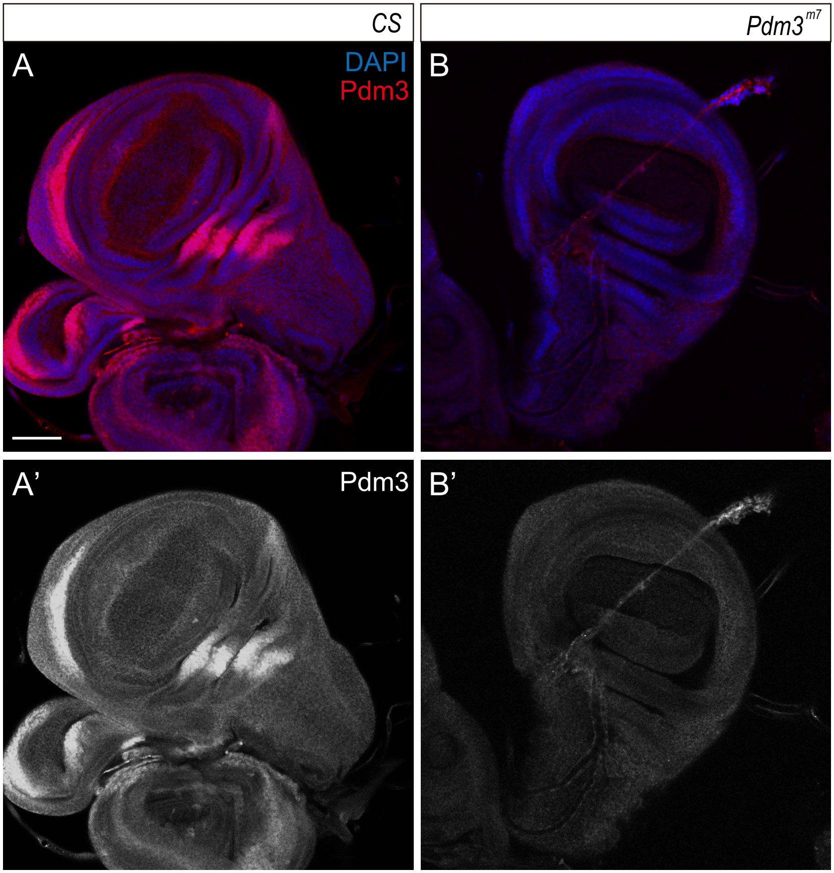

Figure S1. The level of Pdm3 is extremely low in *pdm3^{m7}* wing discs.

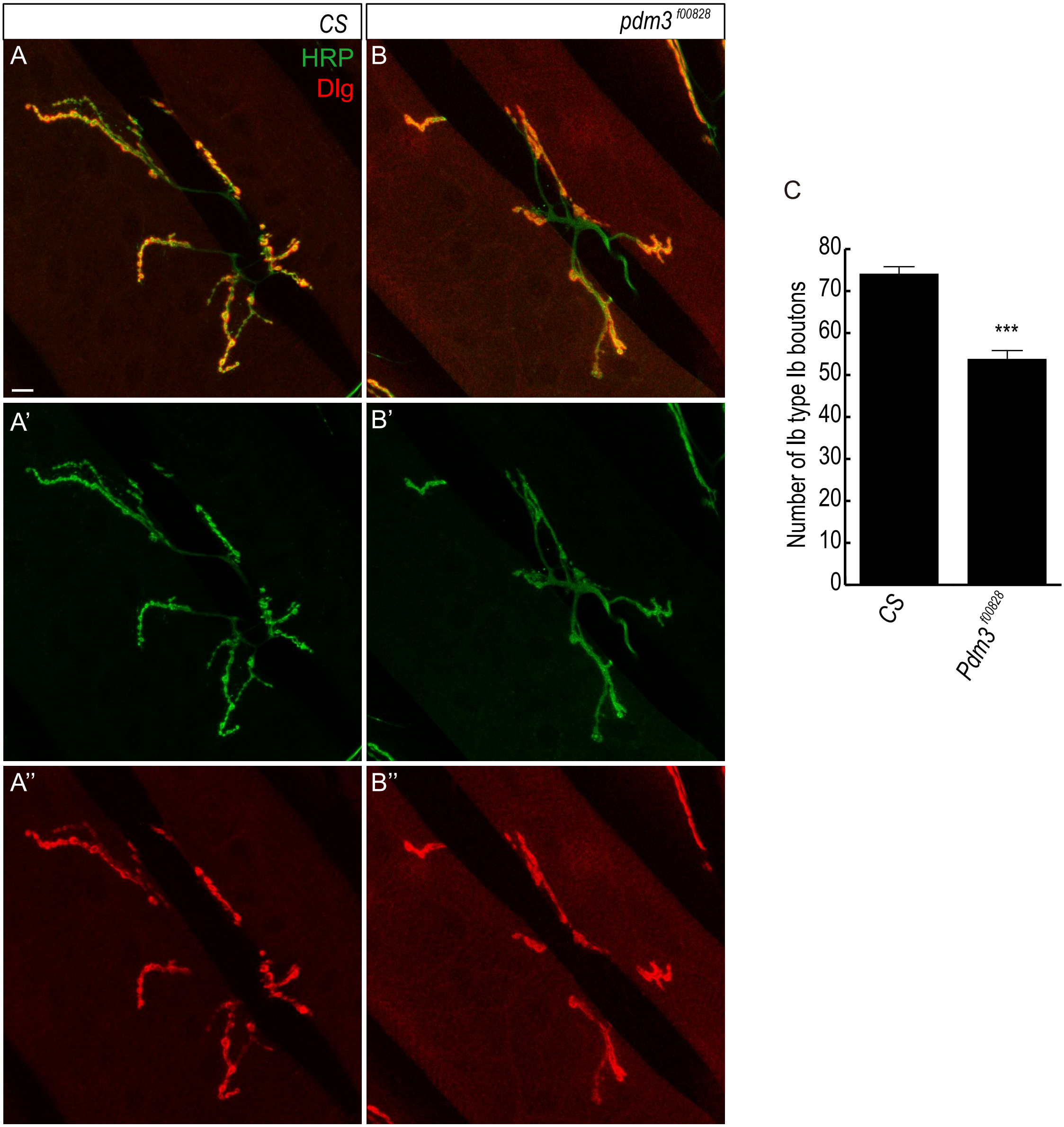

CS
pdm3 f00828
A
HRP
Dlg
B
A'
B'
A''
B''
C
Number of Ib type Ib boutons
80
70
60
50
40
30
20
10
0
***
CS
Pdm3 f00828

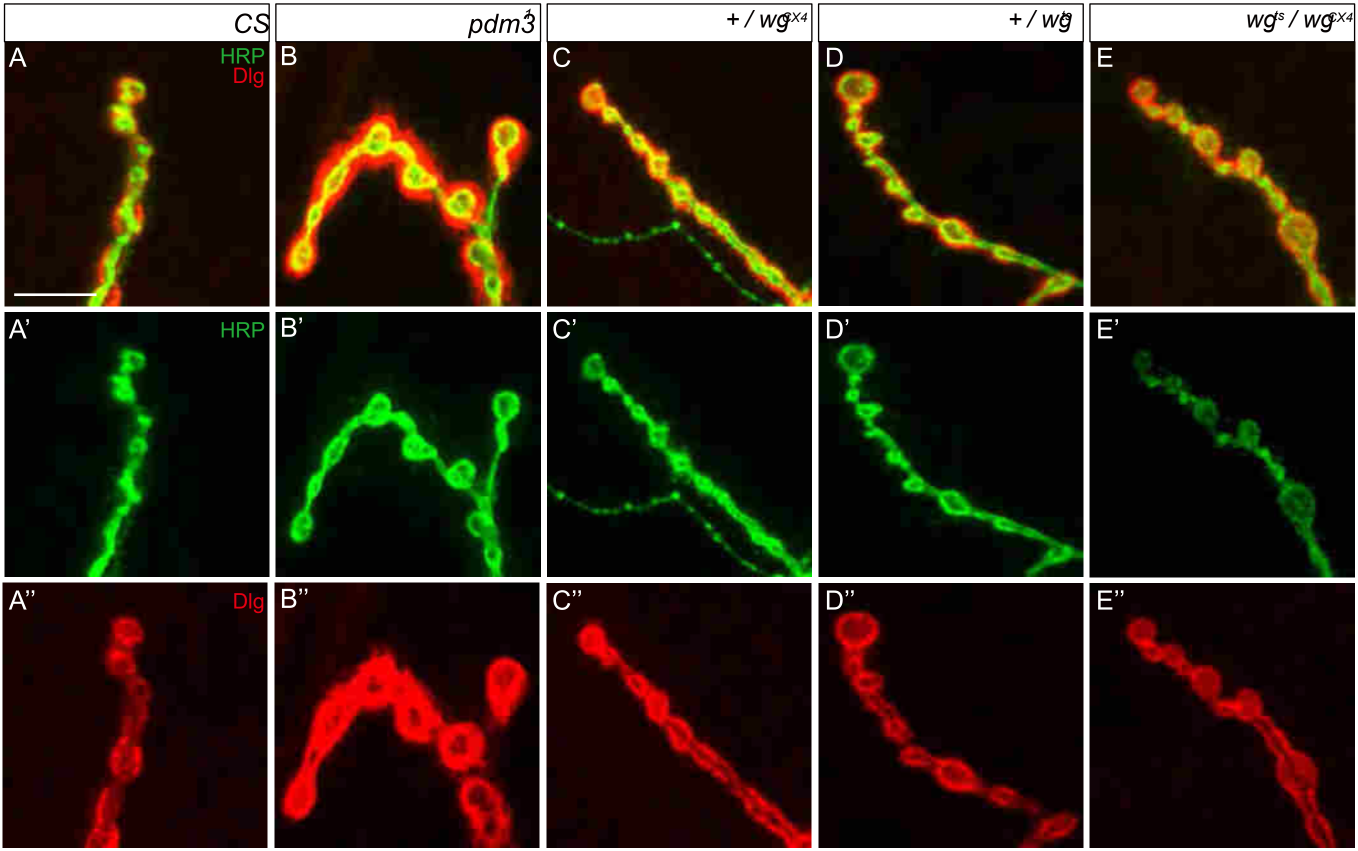

Figure S3. The level of Dlg is increased in *pdm3¹* NMJs.

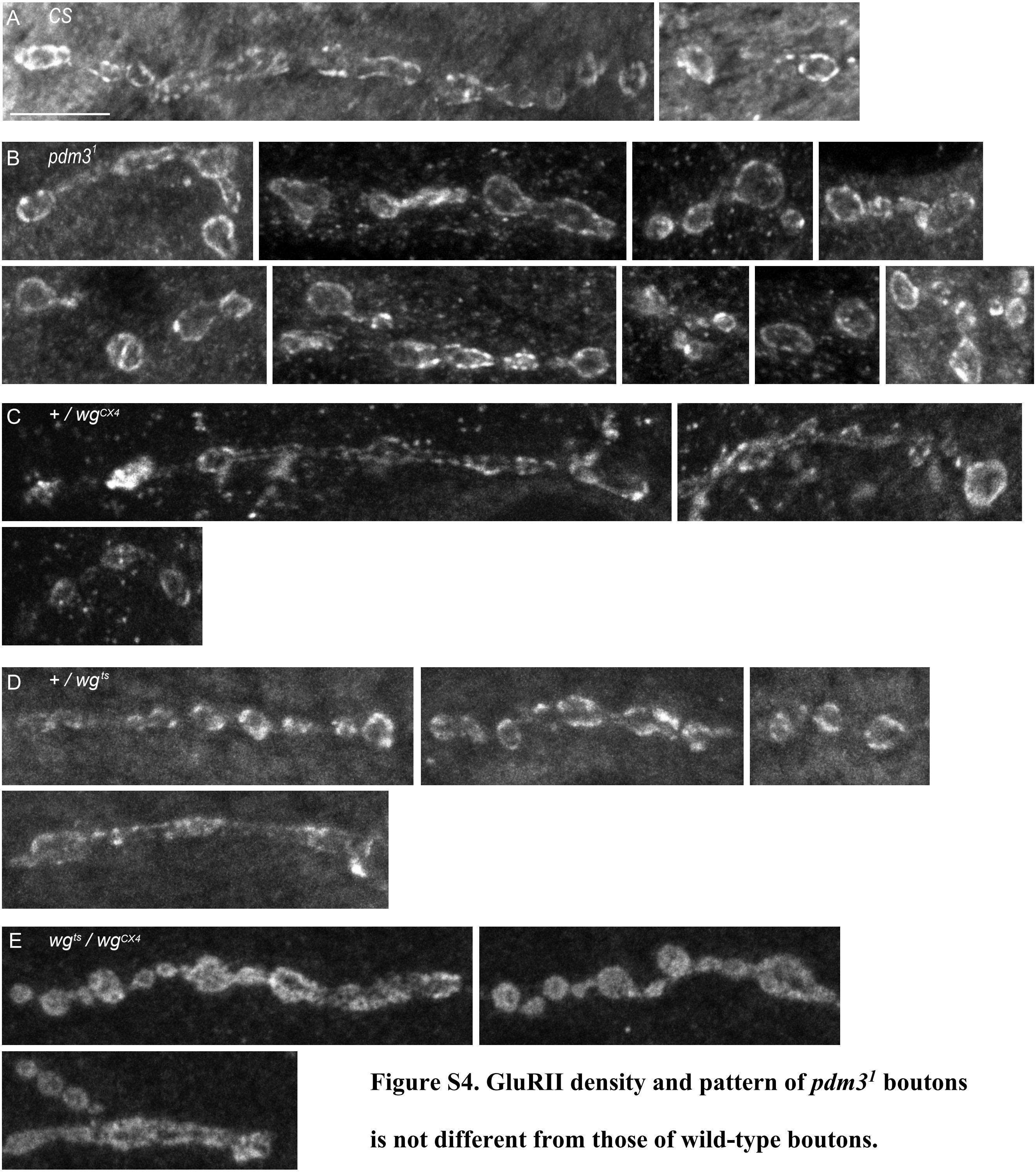

Figure S4. GluRII density and pattern of *pdm3[1]* boutons is not different from those of wild-type boutons.

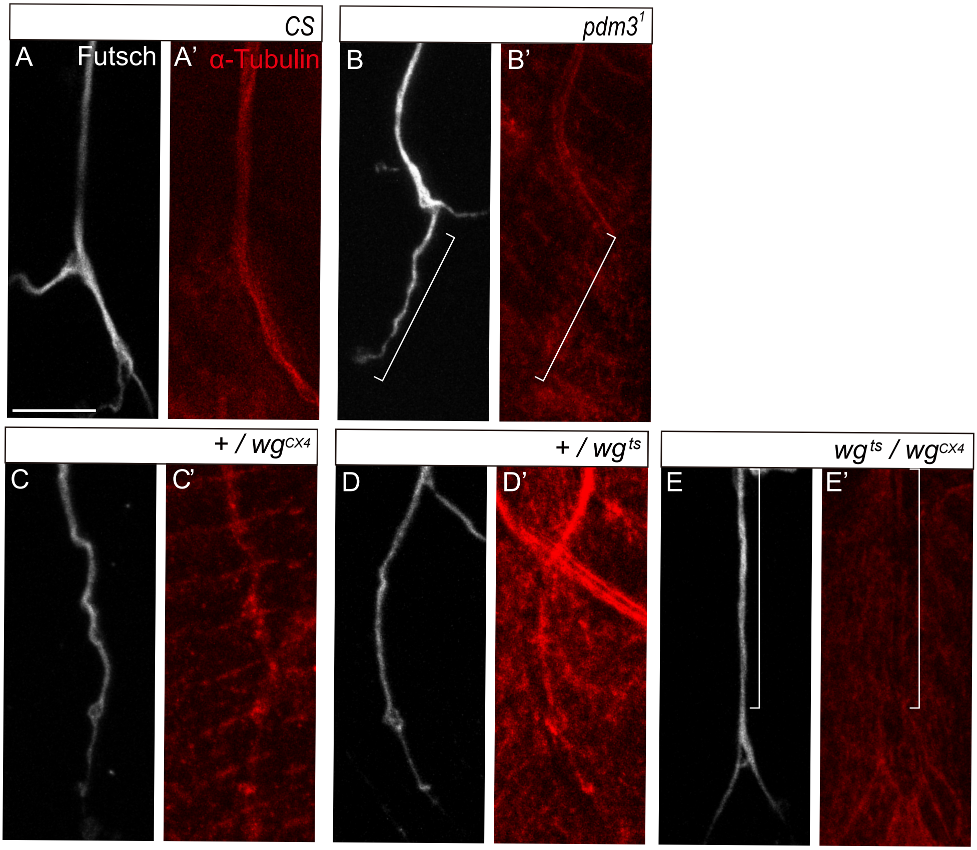

Figure S5. Pdm3 and Wg are required for stabilization of microtubules.

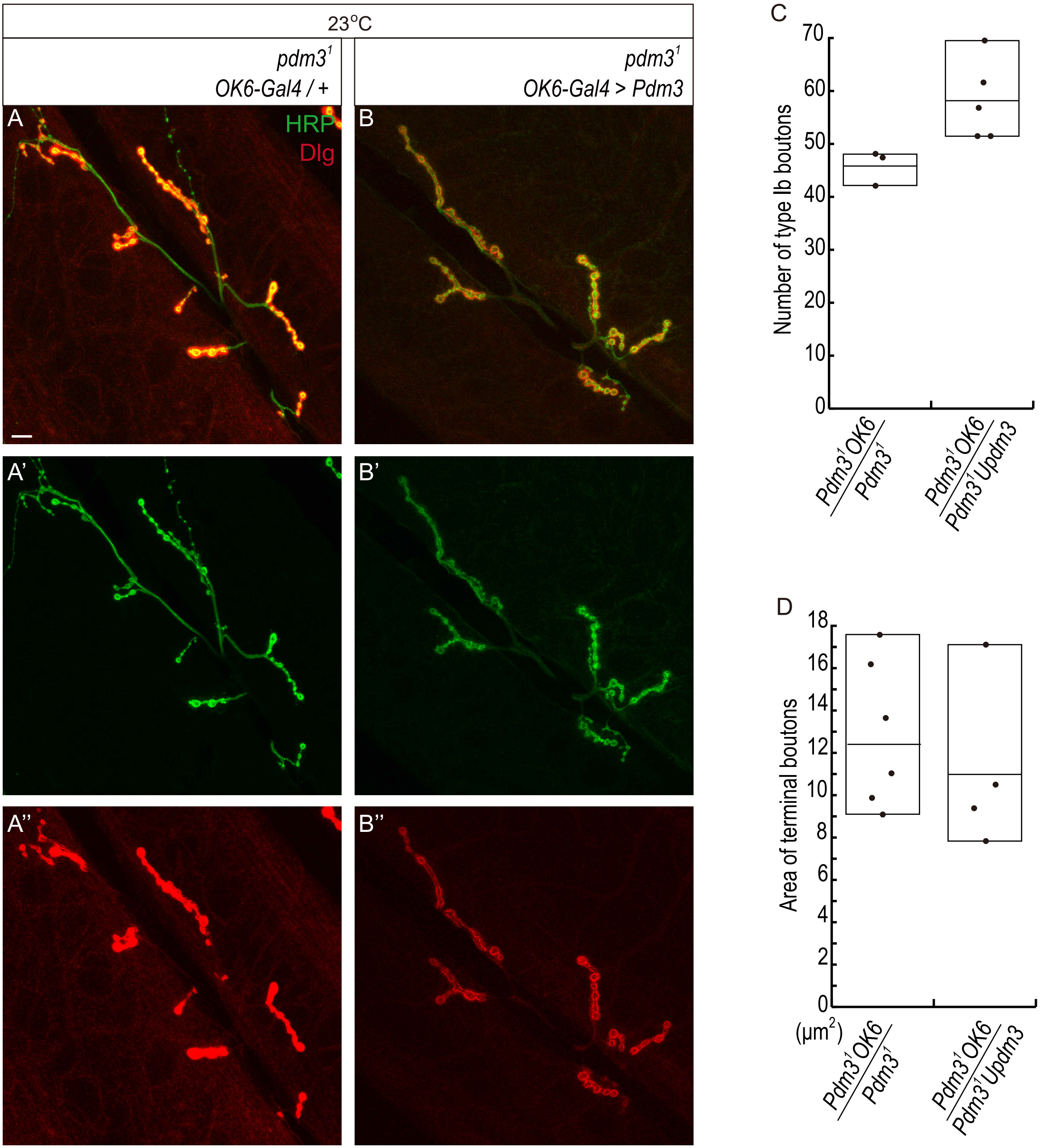

Figure S6. Bouton phenotype of *pdm3¹* NMJs is rescued by Pdm3 overexpression by *OK6-Gal4*.

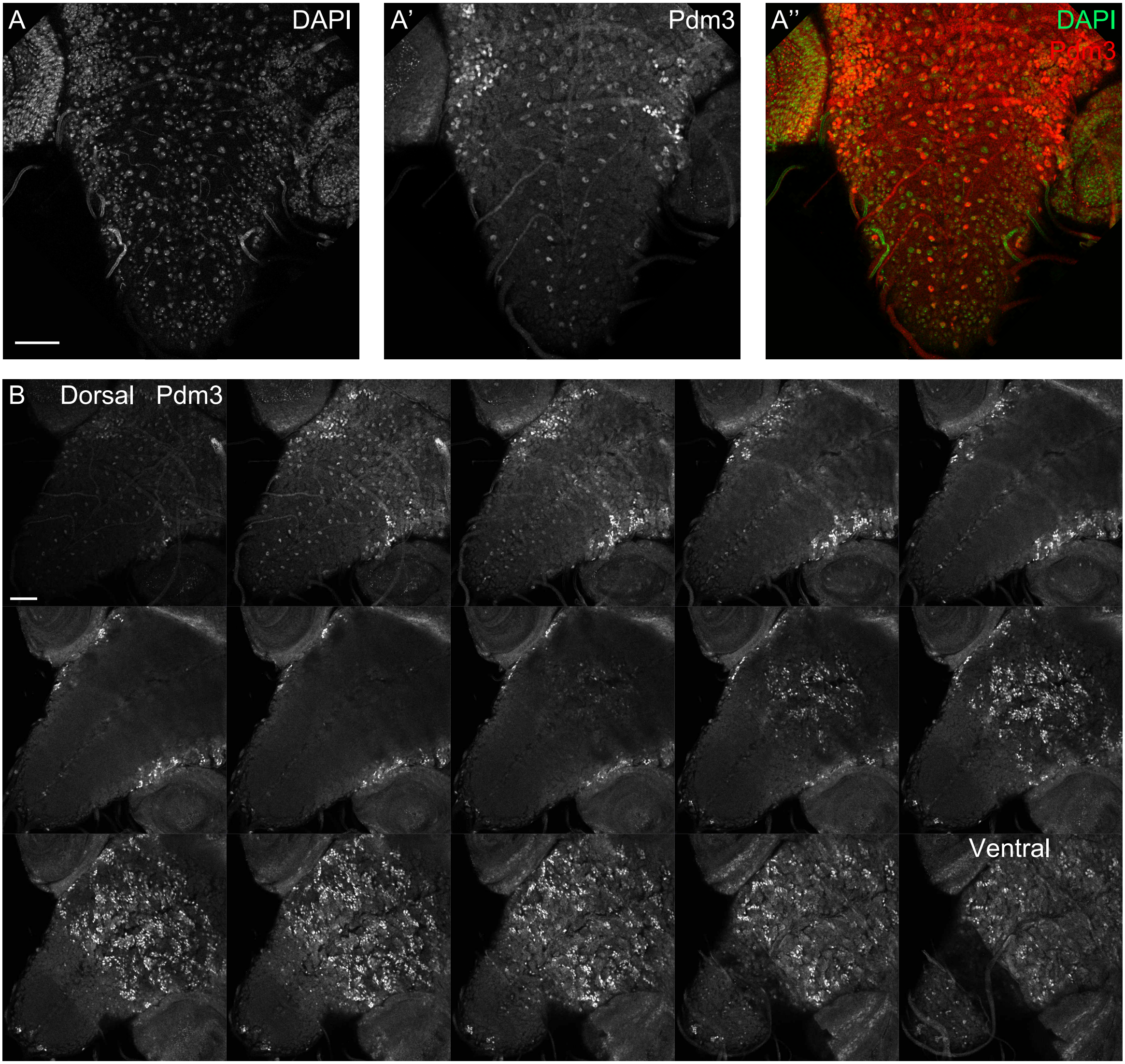

Figure S7. Pdm3 expression is more prominent in the anterior than the posterior region of ventral ganglion.

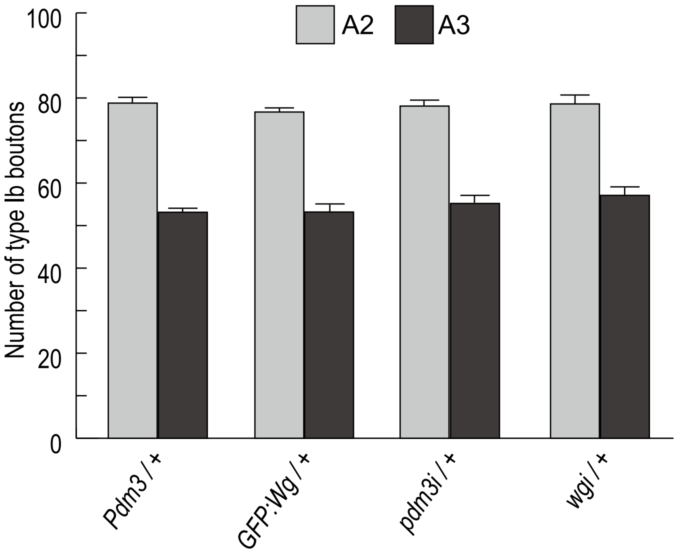

Figure S8. *UAS* lines as controls show similar number of boutons to wild-type.

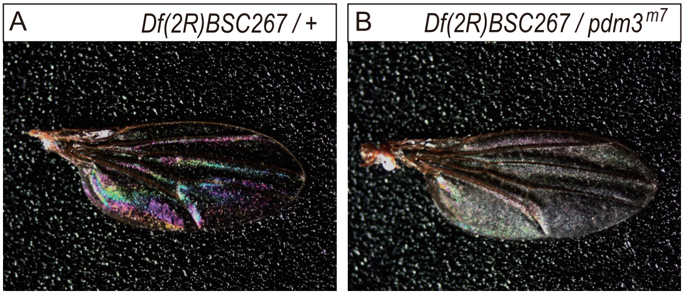

Figure S9. Wing blades are detached in *pdm3* wings.

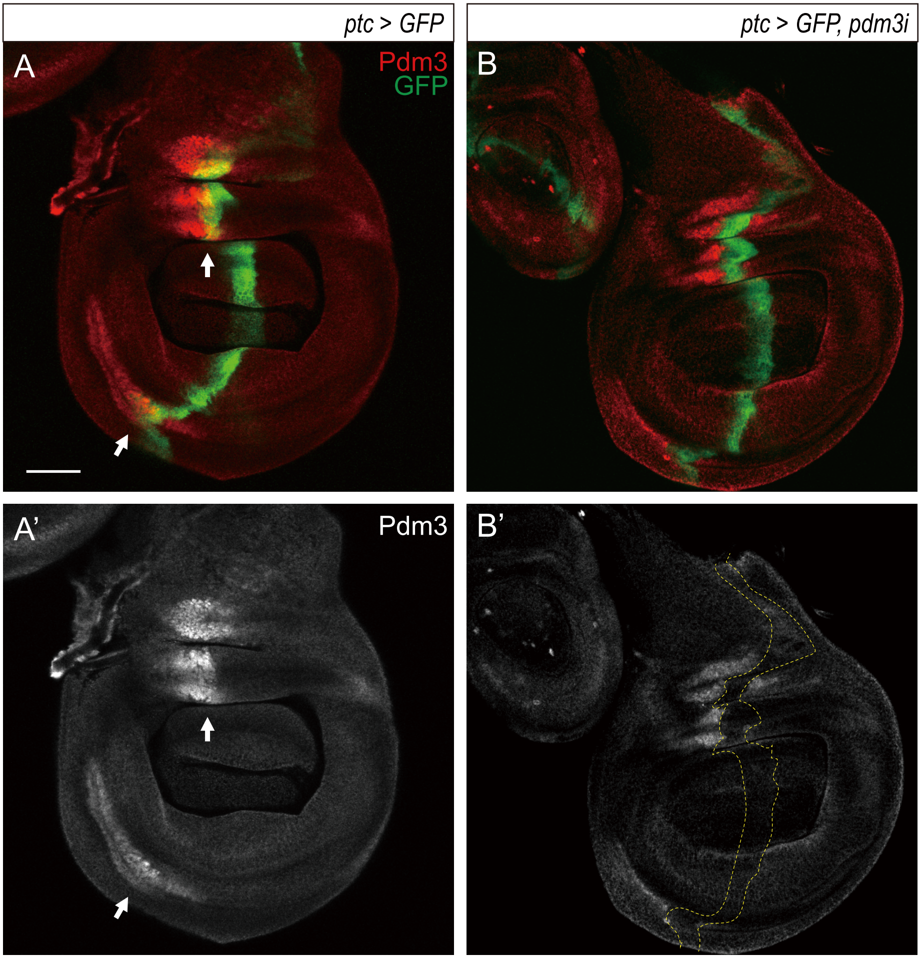

Figure S10. ***pdm3 RNAi*** **is functional.**

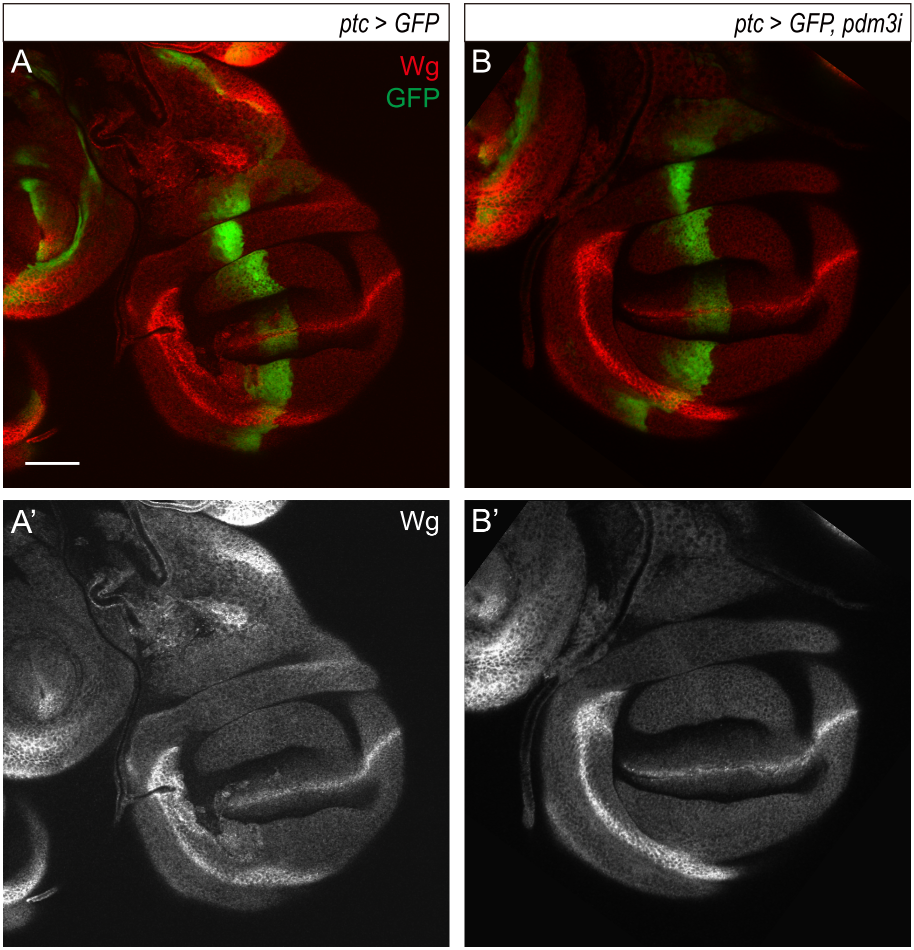

Figure S11. ***pdm3 RNAi*** **does not change expression pattern of Wg in wing discs.**

	phenotypes			
genoypes	defects in locomotion	wing		
		PCP	wing drooping	incomplete adhesion
$\frac{Df(2R)Exel6058}{Df(2R)BSC263}$	+	+	+	+
$\frac{pdm3^{m7}}{Df(2R)BSC263}$	+	+	+	+
$\frac{pdm3^{m7}}{pdm3^{f00828}}$	+	+	+	+
$\frac{pdm3^{1}}{pdm3^{f00828}}$	+	+	+	+
$\frac{pdm3^{m7}}{pdm3^{m7}}$	+	+	+	+
$\frac{pdm3^{f00828}}{pdm3^{f00828}}$	+	+	+	+
$\frac{pdm3^{1}}{pdm3^{1}}$	+	+	+	+

Table 1. Defects in wings and locomotion of adult flies in various combinations of *pdm3* alleles

Gal4s	phenotypes
ap-Gal4	Embryonic lethality 100%
en-Gal4	Embryonic lethality 95%
ptc-Gal4	Embryonic lethality 100%
nub-Gal4	Pupal lethality > 95%, No wing
GMR-Gal4	Pupal lethality > 95%, Small eyes
sona-Gal4	Pupal lethality 100%
30A-Gal4	Pupal lethality 100%
24B-Gal4	Pupal lethality 100%

Table 2. Lethal phenotype by overexpressed Pdm3

Figure S1. The level of Pdm3 is extremely low in *pdm3*^{m7} wing discs.

(**A, B**) Wing discs of control (A) and *pdm3*^{m7} mutant (B). Scale bars: 10 μm.

Figure S2. NMJs of *pdm3*^{f00828} exhibit reduction in bouton number.

(**A, B**) NMJs of control (A) and *pdm3*^{f00828} mutant (B). (**C**) The bar graph shows number of boutons in control and *pdm3*^{f00828} mutant. Data are represented as mean ±SEM. Scale bars: 10 μm.

Figure S3. The level of Dlg is increased in *pdm3*^{1} NMJs.

(**A-E**) *CS, pdm3*^{1}, *wg*^{ts}/+, *wg*^{cx4}/+, and *wg*^{ts}/*wg*^{cx4} NMJs were stained for Dlg and HRP. Only *pdm3*^{1} boutons have high level of Dlg (B"). Scale bars: 10 μm.

Figure S4. GluRII density and pattern of *pdm3*^{1} boutons

is not different from those of wild-type boutons.

(**A-E**) The GluRII staining of NMJs in muscle 4 of the A2 segment in *CS, pdm3*^{1} and *wg* mutants. The pattern and density of GluRII is not altered by loss of Pdm3 (A,B) but the cluster-like GluRII pattern disappears in *wg*^{ts}/*wg*^{cx4} boutons compared to controls (C-E). Scale bars: 10 μm.

Figure S5. Pdm3 and Wg are required for stabilization of microtubules.

The NMJs in muscle 4 of the A2 segment in *pdm3*^{1} and *wg*^{ts}/*wg*^{cx4} mutants. (**A, B**) Futsch and α-Tubulin in control (A) and *pdm3*^{1} (B). White brackets mark regions stained for Futsch but not for α-Tubulin. (**C-E**) Both Futsch and α-Tubulin signals are detected in controls (C,D) but α-Tubulin signal is extremely low in *wg*^{ts}/*wg*^{cx4} (E). Scale bars: 10 μm.

Figure S6. Bouton phenotype of *pdm3*^{1} NMJs is rescued by Pdm3 overexpression by *OK6-Gal4*.

UAS-pdm3 pdm3^1 */OK6-Gal4 pdm3*^{1} larvae were cultured at 23°C due to lethality by Pdm3 overexpression and *pdm3*^{1} mutant cold-sensitivity. (**A,B**) NMJs of *pdm3*^{1} *OK6-Gal4/pdm3*^{1} larvae (A) and *UAS-pdm3 pdm3*^{1} */OK6-Gal4 pdm3*^{1} larvae (B) stained for HRP and Dlg. The level of Dlg is abnormally high in (A") but rescued to normal level in (B"). (**C,D**) Number (C) and size (D) of type Ib boutons in NMJs of *pdm3*^{1} *OK6-Gal4/pdm3*^{1} and *UAS-pdm3 pdm3*^{1} */OK6-Gal4 pdm3*^{1} larvae. n = 3 for formal, 5 for latter. Scale bars: 10 µm.

Figure S7. Pdm3 expression is more prominent in the anterior than the posterior region of ventral ganglion.

(**A**) DAPI and Pdm3 expression in the second dorsal section of basal ganglion in (B). (**B**) Serial z-sections of ventral ganglion from dorsal to ventral region for Pdm3. Scale bars: 10 µm.

Figure S8. *UAS* lines as controls show similar number of boutons to wild-type.

The number of boutons in muscle 6/7 of A2 and A3 in all *UAS* lines in this study. Data represented as mean ±SEM.

Figure S9. Wing blades are detached in *pdm3* wings.

Transparent appearance of + */Df(2R)BSC267* wing (**A**) indicates attachment of dorsal and ventral wing blades. In contrast, opaque appearance of *pdm3*^{m7}*/Df(2R)BSC267* wing (**B**) indicates detachment of the two wing blades.

Figure S10. *pdm3 RNAi* is functional.

(**A**) Localization pattern of Pdm3 in the wing discs of late 3rd instar larvae. Pdm3 is highly

expressed in the presumptive hinge region. The localization of Pdm3 is somewhat overlapping with *ptc-Gal4* expression region in the presumptive hinge (arrows in A and A'). (**B**) *ptc>pdm3 RNAi* reduced the level of Pdm3 protein in the *ptc* region outlined in B'. Scale bars: 10 µm.

Figure S11. *pdm3 RNAi* does not change expression pattern of Wg in wing discs.

(**A**) Wg (red) in *ptc>GFP* wing disc. (**B**) Wg (red) in *ptc>pdm3i, GFP* wing disc. Wg patterns in (A) and (B) are shown in black and white in A' and B'. Scale bars: 10 µm.

Table 1. Defects in wings and locomotion of adult flies in various combinations of *pdm3* alleles.

Flies with the genotypes listed show identical defects in locomotion and wings.

Table 2. Lethal phenotype by overexpressed Pdm3.

Overexpression of Pdm3 causes lethality with all *Gal4* lines tested.

Movie 1. Loss of *pdm3* causes locomotion defect in adults.

The movie shows control *+/Df(2R)BSC267* flies and $pdm3^{f00828}$/ *Df(2R)BSC267* transheterozygotes. All flies were 1 to 3 days old.
